# Appropriate Image Selection With Virtual Reality in Vestibular Rehabilitation: Cross-sectional Study

**DOI:** 10.2196/40806

**Published:** 2023-04-13

**Authors:** Kerem Ersin, Emre Gürlek, Hakan Güler, Çiğdem Kalaycık Ertugay, Mustafa Bülent Şerbetçioğlu

**Affiliations:** 1 Graduate School of Health Sciences Istanbul Medipol University Istanbul Turkey; 2 Department of Audiology Istanbul Education Research Hospital Istanbul Turkey; 3 Faculty of Health Sciences Istanbul Medipol University Istanbul Turkey

**Keywords:** balance, computerized dynamic posturography, stress, vestibular rehabilitation, vestibular system, virtual reality

## Abstract

**Background:**

While vestibular rehabilitation with virtual reality (VR) is becoming more popular every day, the disadvantages of this method are not yet clear.

**Objective:**

The aim of this study is to examine the effect of the image to be used in vestibular rehabilitation with VR on the systems that provide body posture.

**Methods:**

The study was carried out with 36 participants (18 women and 18 men) aged 18 to 30 years. To assess balance control components separately, a sensory organization test was administered to the participants in the presence of stressful and relaxing environment images with VR technology. The State-Trait Anxiety Inventory survey was also used to measure the stress values in the created environments.

**Results:**

The State-Trait Anxiety Inventory survey revealed that while stressful videos significantly increased stress, relaxing videos reduced stress. Among measurements obtained in the presence of VR, significant decreases were observed mostly in the visual system data. A significant increase in vestibular system data (*P*=.01) was observed with a decrease in visual system data (*P*<.001) when the relaxing image was presented. Additionally, there was a significant difference in the somatosensory (*P*=.001), composite (*P*=.002), and visual system (*P*<.001) data in the presence of stressful videos.

**Conclusions:**

Although the use of a VR system for vestibular rehabilitation is relatively new, no extant studies have examined how the image type used in VR can affect the integration of visual system data. Therefore, this study is unique in terms of showing the effects of the stress created by the change in the type of the image used in VR. When VR technology is used for therapeutic vestibular rehabilitation for patients whose balance disorder is due to the vestibular system, stress-free videos should be used. However, the use of stressful videos in VR technology will be beneficial in the rehabilitation of those with balance disorders due to the somatosensory system.

## Introduction

Maintaining posture uses a complex mechanism involving the brain’s processing of multiple sensory inputs from the visual, proprioceptive, and vestibular systems by the central nervous system [1]. Computed dynamic posturography (CDP), which is used to examine the effects of these multisensor inputs separately, is a kind of test that can be used to evaluate the mechanisms that ensure the protection of the individual’s posture by using different test positions similar to those we often experience in our daily lives [2]. The sensory organization test (SOT), one of the CDP test protocols, is a test that can evaluate a person’s ability to use inputs from the vestibular, somatosensory, and visual systems to maintain balance in different stimulus situations and to monitor the compensatory condition in case of balance deterioration [3].

Virtual reality (VR) can be defined as a computer-generated simulation of a 3D image or environment with which the user can interact. Recently, the scope of VR use has expanded greatly, and it is being used in many areas, especially in rehabilitation programs, where it is increasing the effectiveness of vestibular rehabilitation. Studies have shown that a VR protocol may be a safe option to improve postural control and quality of life in individuals with vestibular disorders [4-6].

The classical stress response is a negative feedback system mediated by the hypothalamic-pituitary-adrenaline axis [7]. Studies have found that balance is affected in people who are under stress [8-10]. According to Saman et al [11], stress may affect central vestibular function either directly through the effects of cortisol and corticosteroids on ion channels and neurotransmission in the brain or indirectly through stress-related neuroactive substances. Gliddon et al [12] observed a significant increase in postoperative salivary cortisol levels in guinea pigs undergoing unilateral vestibular deafferentation.

One of the most widely used clinical tests for the measurement of anxiety is the State-Trait Anxiety Inventory (STAI) survey (Multimedia Appendices 1 and 2). The survey test, which evaluates stress immediately after an individual has undergone a test in a research or clinical setting, was administered, and the Turkish validity form, the reliability of which had been ascertained before, was filled in. There are questions in the inventory that increase and decrease the anxiety score [13,14].

Additionally, in the study by Seinfeld et al [15], increases in stress indicators (heart rate and skin responses) were observed in stress environments created by a VR system [16]. However, in some other published studies, the effect of stress was not considered in vestibular rehabilitation using a VR system. For this reason, the aim of this study is to examine the effect of the image to be used in vestibular rehabilitation with VR on the vision-proprioception-vestibular systems that provide body posture.

## Methods

### Participants and Recruitment

We calculated the sample size using power analysis. In the study, we set an α (type I error) of 5%, Cohen medium standardized effect size of 0.5, and power of 80% (with β [type II error] set to 20%). Power analysis revealed that a minimum of 28 patients were required. A total of 36 patients, 18 men (aged 23.33, SD 2.54 years) and 18 women (aged 21.66, SD 2.22 years) between the ages of 18 and 30 years (sample mean age 22.50, SD 2.50 years), were included in the final analysis. While individuals who were psychologically healthy and had no problems with balance in daily life in the preceding 1-year period were included in the study, those with eye problems, a fear of heights, dizziness in daily life, physical discomfort, or regular use of alcohol or drugs were excluded from the study.

### Experimental Design

The study was conducted in the audiology clinic at the Istanbul Training and Research Hospital between February 23 and May 10, 2021. A NeuroCom device (Natus Medical Inc) was used to administer the SOT from among the CDP test repertoire.

The SOT measures how well the patient maintains equilibrium under 6 sensory conditions. Each condition is scored between 1 and 100, with an increased score indicating better stability. Each of the 6 conditions is repeated 3 times, and the duration of each test is 20 seconds. After 6 conditions (ie, 18 tests) the composite equilibrium score is then determined [16].

For the first 3 conditions, the force plates are fixed, and for the other 3 conditions, they move in anterior and posterior directions. In the first condition (baseline), the person is placed on the system such that all sensory information involved in postural control is available. In the second condition, the participant is tested with eyes closed (the visual information system is eliminated). In the third condition, the person’s eyes are open, but the visual environment moves to present incorrect visual sequences. In the fourth to sixth conditions, the force plates are moved so that they act as what is referred to as a “sway referenced surface,” making proprioceptive information inaccurate; hence, the subjects use visual and vestibular (or only vestibular) information to maintain their balance, in addition to having to overcome the inaccurate proprioception signals by relying on other systems and compensatory mechanisms. In the fourth condition, information from the visual and vestibular systems is evaluated. In the fifth condition, the eyes are closed. In this condition, information from the vestibular system is evaluated. In the sixth condition, inappropriate visual arrays are presented; thus, information from the vestibular system is evaluated [16].

Comparison of scores from the first 2 trials produces a “somatosensory score”; comparison of the score for test 4 with that from test 1 yields the “visual score”; comparison of scores from trial 5 to trial 1 reflects the “vestibular score.” Comparing the sum of scores from the third and the sixth trials with the sum of scores from the second and fifth trials yields the “visual preference score” [17].

**Figure 1:**
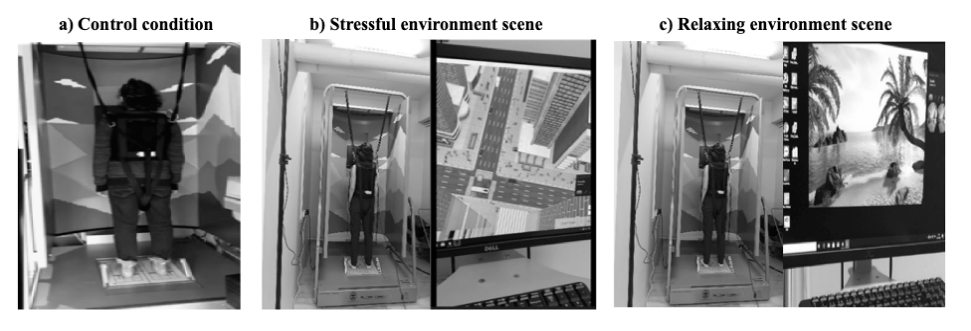
Three different visually generated test environments.

The SOT, one of the CDP tests, was administered to the participants in 3 different conditions generated using VR: in the control condition, in a stressful environment, and in a relaxing environment (Figure 1). An HTC VIVE VR system was used to generate the different environments. In the control condition, normalization values were determined by administering the SOT to the participants without wearing VR glasses (Figure 1).

To generate a stressful environment in the VR simulation, participants were raised to a height of 40 meters in a simulation of an elevator. Then, in the simulation, the elevator door opened, and they walked onto a platform that appeared in front of them. The end of the platform was mapped to the test area of the CDP device. When they arrived at this position, the SOT was administered to the participants while they saw VR images from a simulated height of 40 meters (Figure 1).

In the relaxing environment, while participants were waiting for the test on the CDP device, a meditation application was opened in VR. The SOT was administered while the participants were in the relaxing scenery (Figure 1).

Considering that the participants could adapt to the test, the tests were applied to each participant starting from a different condition. This aimed at preventing possible adaptation. A participant who completed one condition rested for at least 1 hour, after which another condition was applied.

After the tests, the participants took the STAI survey. Certain questions in the survey could increase or decrease the anxiety score. For each question, a score between 1 (or –1) and 4 (or –4) is assigned in accordance with the positive or negative characteristics of the question. The highest score is 80 and the lowest score is 20. The higher the total anxiety score, the higher the anxiety level of the person taking the survey [13].

### Ethics Approval

The study was approved by the Non-Invasive Clinical Research Ethics Committee of Istanbul Medipol University (923/2020). All individuals participating in the study received explanations of the aims of the study, how long it would last, and practices and expectations. Participants then signed the consent form.

### Statistical Analysis

The minimum subject size was estimated using G*Power (version 3.1; Heinrich-Heine-Universität Düsseldorf). The data analysis of our study was performed using SPSS (version 22.0; SPSS Inc). Descriptive statistics included mean and SD values. Normality of the distribution and homogeneity of the data were analyzed with the Kolmogorov-Smirnov test. A triple comparison of the groups (control condition [CC], stressful condition [SC], and relaxing condition [RC]) was carried out using the Friedman test. CC-RC, CC-SC, and RC-SC comparisons were made using the Wilcoxon signed-rank test. The statistical significance level was set to a *P* value of ≤.05 when analyzing the results of the Wilcoxon signed-rank test. In the Friedman test, statistical significance level was set to a *P* value of ≤.017.

## Results

### Triple Comparisons

**Figure 2:**
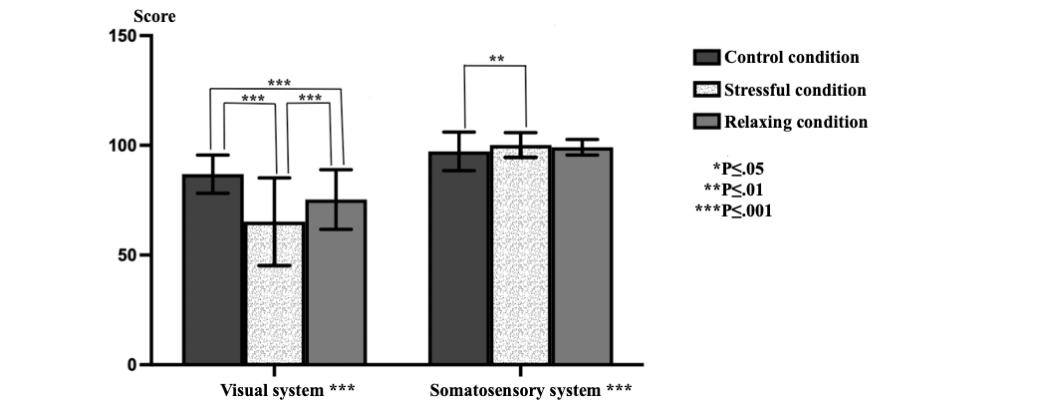
The sensory organization test and visual and somatosensory system data.

**Figure 3:**
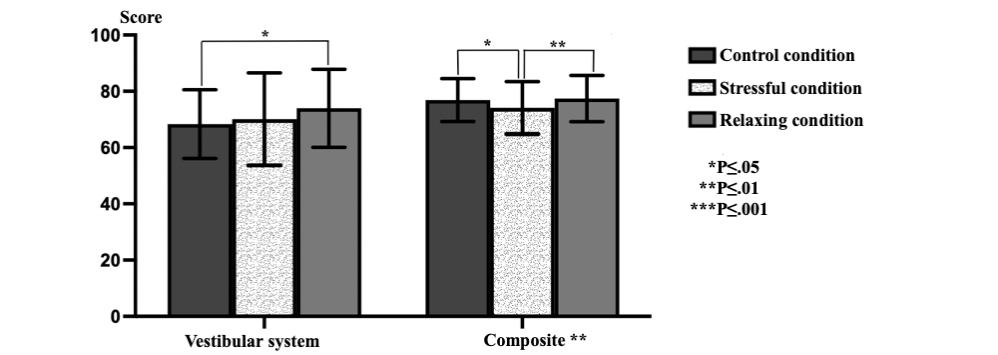
The sensory organization test for the vestibular system and composite data.

**Figure 4:**
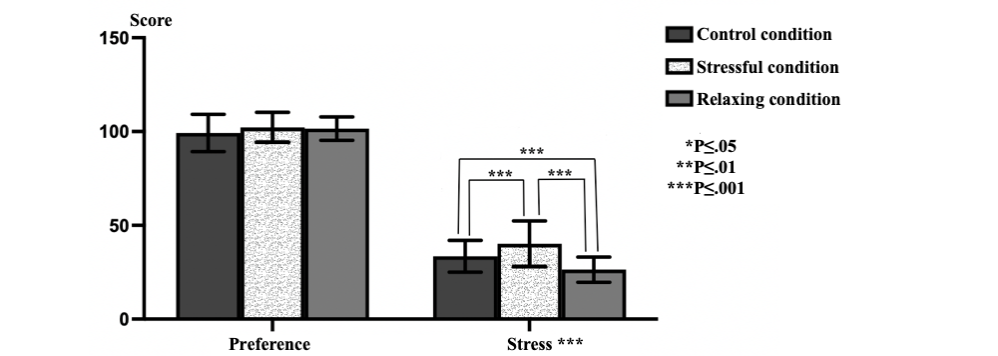
The sensory organization test, preference scores, and survey stress data.

Significant differences were observed in the visual system (*P*<.001) and somatosensory system (*P*=.001) data in the triple comparison of SOT data obtained in different stress environments (Figure 2). While no significant difference was observed in the vestibular system (*P*=.03) data, a significant difference was observed in the composite (*P*=.002) values (Figure 3). While a significant difference was observed in stress values (*P*<.001), there was no significant difference in the preference values (Figure 4).

### Dual Comparisons

Significant differences were observed in visual system data in pairwise comparisons between the cases (CC-RC and CC-SC, *P*<.001; RC-SC, *P*=.001). A significant difference was observed when comparing the CC and SC environments (*P*=.002) in the somatosensory system data. A significant difference was observed when comparing the CC and RC environments (*P*=.01) in the vestibular system data (Table 1). No significant difference was observed in the comparisons made with preference data. A significant difference was observed when comparing the CC and SC environments (*P*=. 02) and the SC and RC environments (*P*=.005) in the composite data. When only the stress data were evaluated, a significant difference was observed when comparing the CC and SC environments (*P*<.001), CC and RC environments (*P*<.001), and SC and RC environments (*P*<.001; Table 2).

**Table 1:** Dual and triple comparisons of visual, vestibular, and somatosensory system data in 3 different cases.

	Control condition (CC), mean (SD)	Stressed condition (SC), mean (SD)	Relaxed condition (RC), mean (SD)	*P* value (CC-SC-RC)	*P* value (CC-SC)	*P* value (CC-RC)	*P* value (SC-RC)
Visual system	86.88 (8.70)	65.22 (19.98)	75.30 (13.61)	<.001	<.001	<.001	.001
Vestibular system	68.36 (12.20)	70.11 (16.46)	73.97 (13.87)	.03	.43	.01	.12
Somatosensory system	97.22 (8.79)	100.16 (5.64)	99.11 (3.52)	.001	.002	.21	.10

**Table 2:** Dual and triple comparisons of preference, composite, and stress data in 3 different cases.

	Control condition (CC), mean (SD)	Stressed condition (SC), mean (SD)	Relaxed condition (RC), mean (SD)	*P* value (CC-SC-RC)	*P* value (CC-SC)	*P* value (CC-RC)	*P* value (SC-RC)
Preference	99.19 (9.95)	102.25 (8.03)	101.55 (6.27)	.50	.12	.30	.97
Composite	76.91 (7.62)	74.11 (9.31)	77.44 (8.21)	.002	.02	.52	.005
Stress	33.50 (8.48)	40.16 (12.18)	26.38 (6.72)	<.001	<.001	<.001	<.001

## Discussion

### Principal Findings

The aim of the study was to investigate the effect of stress on the systems that provide data for maintaining posture. The SOT was administered to the study participants in different stress environments generated with VR. The changes in visual, vestibular, and somatosensory systems were examined with the SOT. Our results indicate that stress, especially presented visually, has a significant effect on the systems that protect posture.

Wuehr et al [18] took people to a height of 40 meters with a VR-based elevator simulation and observed specific changes in subjective fear levels, muscle activity, and balance control. In our study, like that of Wuehr et al [18], the SOT was administered by raising people to a height of 40 meters in an elevator simulation with a VR system as an instant stress source. When we examined the change in stress in the participants using the STAI survey before and after the test, we found that the participants were affected by stress in the presence of a stressful video. In the presence of a relaxing video, we found that the stress level was significantly lower than in the control group. Thus, we proved that our image selection is effective for assessing stress factors.

VR, which we use as an instant stress generation tool, has been used frequently in clinical studies with the development of technology in recent years [19]. In addition, its use in vestibular rehabilitation is increasing [20]. It has not been specifically reported in the literature whether VR has an adverse effect in VR-based vestibular rehabilitation or whether it has an adverse effect on the systems that provide data for maintaining posture [21]. Micarelli et al [22,23] reported that symptoms of nausea, oculomotor stress, and disorientation decreased over time in people participating in a vestibular rehabilitation program with VR. Both studies saw a significant reduction in nausea, oculomotor stress, and disorientation in the first to fourth week of the rehabilitation program with VR [22,23]. However, the instantaneous effect of VR and the effect of the video type used on treatment were not investigated in these studies. In our study, when we looked at the instantaneous effect of VR on the systems that maintain posture by comparing the CC and RC environments and considering the results of the SOT, we found that VR application does not directly cause stress, but rather makes the person feel more comfortable than normal. However, we also observed that VR had a negative effect on the visual system while in this condition. Although there was no negative effect on the somatosensory system, we observed a significant increase in the values of the vestibular system. With the decrease in values for the visual system and the increase in those for the vestibular system, there was no significant decrease in the composite value, which is the general balance score. We believe that VR-based vestibular rehabilitation will be a successful process based on these findings. To avoid potential side effects from staying in a virtual environment for a long time during this process, special attention should be paid to the effect on the visual system, cyber sickness, dizziness under visual stimuli, nausea, and imbalance [24,25].

In Öztürk’s [25] study, individuals were evaluated with cervical vestibular-evoked myogenic potentials and video head impulse tests in the presence of visual illusions. The amplitudes of the vestibular-evoked myogenic potentials increased in cases where visual illusions were presented, and gains in some semicircular canals increased significantly. This showed that the vestibular system works more efficiently when there is a decrease in the consistency of the data obtained from the visual system [25]. In this study, the increase in vestibular system data is compatible with the visual stimulus provided by wearing the VR device.

In a study by Smyth et al [26] , healthy individuals with the highest cortisol response to psychosocial stress—that is, those who were most affected—had less-impaired postural sway when exposed to visual movement [26]. This is consistent with the findings of our study, in which the somatosensory system performed better when the VR image containing the stress stimulus was used, because in healthy people, minor oscillations are modulated by the ankle, which is part of the somatosensory system.

Psychological effects are common in patients experiencing vertigo or dizziness. In particular, the fear of falling and the resulting stress and anxiety seriously impair the quality of life of these individuals [27]. Eagger et al [28] found that patients with vestibular symptoms had higher social stress and trait anxiety scores than those without vestibular symptoms. The balance-stress neuroanatomical link has been defined as follows: the nucleus tractus solitarius has been shown to have extensive connections with the vestibular nuclei, both directly and through indirect projections through the parabrachial nucleus, which provides major inputs to the limbic system, including the enlarged central amygdaloid nucleus, infralimbic cortex, and hypothalamus [29]. Because of this connection between the vestibular system and the limbic system, the stress factor should be taken into account when planning treatment for individuals with vestibular symptoms. Choosing less stressful images during the vestibular rehabilitation phase, especially near the end of the study, will aid in the recovery of vestibular systems.

Studies have shown that stress has an effect on the systems that maintain posture [7,9,11]. In line with the findings of the literature review, this study found that stress has a significant effect on these systems and that the stress effect provided by VR is primarily due to the effect on the visual system. When comparing the RC and SC environments, there was a significant decrease in visual system data and composite scores, accompanied by a significant increase in stress values. When we compared the visual system values of the CC and SC environments, we found a significant decrease as a result of stress, as well as a significant change in stress. Similarly, a significant increase in somatosensory values was observed with stress. This result shows the importance of the image used in vestibular rehabilitation. Confidence in the somatosensory system increased significantly when the participants watched a stressful video, while confidence in the vestibular system increased significantly when a relaxing video was shown. Thus, data on which group would be more effective for use in vestibular rehabilitation with VR, and the appropriate images to be used, should be highlighted.

### Limitations

The SOT is not normally administered while wearing VR glasses. However, to investigate the effects of VR on balance while wearing these glasses, the SOT was used with relaxing images. To investigate the effect of image selection, a stress-inducing image, rather than a relaxing image, was used.

### Conclusions

Our study investigated the effects of stress on the systems that protect posture and how much each of the systems is affected by stress, and the effect on individuals of using VR technology to provoke stress. Our results show that the visually created stressful environment had a significant effect on balance. In individuals with balance disorder, it would be more beneficial to use stressful images if the problem relates to the somatosensory system and to use less stressful images if it is related to the vestibular system. To obtain more successful and quicker results in the treatment processes for individuals with balance problems, it is necessary to focus on the stress condition and its effect on patients. In addition, the decrease in visual system data as a result of the stress we induced visually and the effect of rehabilitation with VR in people whose visual system contributes to their balance disorder should be the subject of further studies.

## Appendix

Multimedia Appendix 1 Stait-Trait Anxiety Inventory.

Multimedia Appendix 2 Stait-Trait Anxiety Inventory (Turkish).

## Abbreviations

CC: control condition
CDP: computed dynamic posturography
RC: relaxing condition
SC: stressful condition
SOT: sensory organization test
STAI: State-Trait Anxiety Inventory
VR: virtual reality

## References

[1] Shumway-Cook A, Horak F B (1986). Assessing the influence of sensory interaction of balance. Suggestion from the field. Phys Ther.

[2] Broglio SP, Sosnoff JJ, Rosengren KS, McShane K (2009). A comparison of balance performance: computerized dynamic posturography and a random motion platform. Arch Phys Med Rehabil.

[3] Marioni G (2015). Balance function assessment and management. Hearing Balance Commun.

[4] Leng Y, Lo WLA, Mao YR, Bian R, Zhao JL, Xu Z, Li L, Huang DF (2022). The impact of cognitive function on virtual reality intervention for upper extremity rehabilitation of patients with subacute stroke: prospective randomized controlled trial with 6-month follow-up. JMIR Serious Games.

[5] Rosiak O, Krajewski K, Woszczak M, Jozefowicz-Korczynska M (2019). Evaluation of the effectiveness of a virtual reality-based exercise program for Unilateral Peripheral Vestibular Deficit. VES.

[6] Park JH, Jeon HJ, Lim E, Koo J, Lee H, Kim H, Lee JS, Song C, Hong SK (2019). Feasibility of eye tracking assisted vestibular rehabilitation strategy using immersive virtual reality. Clin Exp Otorhinolaryngol.

[7] Herman JP, Figueiredo H, Mueller NK, Ulrich-Lai Y, Ostrander MM, Choi DC, Cullinan WE (2003). Central mechanisms of stress integration: hierarchical circuitry controlling hypothalamo-pituitary-adrenocortical responsiveness. Front Neuroendocrinol.

[8] Saman Y, Bamiou DE, Gleeson M, Dutia MB (2012). Interactions between stress and vestibular compensation - a review. Front Neurol.

[9] Abd-Manan F (2000). The effect of induced visual stress on three dimensional perception. Malays J Med Sci.

[10] Serpell Benjamin G, Waddington G, McGrath B, Cook C (2020). Is there a link between stress and cognition, and capacity to execute motor skill?. Med Sci Sports Exerc.

[11] Saman Y, Bamiou DE, Gleeson M, Dutia MB (2012). Interactions between stress and vestibular compensation - a review. Front Neurol.

[12] Gliddon CM, Darlington CL, Smith PF (2003). Activation of the hypothalamic-pituitary-adrenal axis following vestibular deafferentation in pigmented guinea pig. Brain Res.

[13] Ayaz A, Bilgin N, Mollaoğlu N (2017). Dental Anksiyetede Durumluk ve Sürekli Kaygı Ölçeğinin Kullanımı. ADO Klinik Bilimler Dergisi.

[14] Spielberger CD, Gonzalez-Reigosa F, Martinez-Urrutia A, Natalicio LFS, Natalicio DS (1971). The State-Trait Anxiety Inventory. Interam J Psychol.

[15] Seinfeld Sofia, Bergstrom Ilias, Pomes Ausias, Arroyo-Palacios Jorge, Vico Francisco, Slater Mel, Sanchez-Vives Maria V (2015). Influence of music on anxiety induced by fear of heights in virtual reality. Front Psychol.

[16] Shams Amir, Vameghi Roshanak, Shamsipour Dehkordi Parvaneh, Allafan Nahid, Bayati Mahdi (2020). The development of postural control among children: repeatability and normative data for computerized dynamic posturography system. Gait Posture.

[17] Nashner LM, Jacobson GP, Newman CW, Kartush JM (1993). Computerized dynamic posturography: clinical applications. Handbook of Balance Function Testing.

[18] Wuehr M, Kugler G, Schniepp R, Eckl M, Pradhan C, Jahn K, Huppert D, Brandt T (2014). Balance control and anti-gravity muscle activity during the experience of fear at heights. Physiol Rep.

[19] Maples-Keller JL, Yasinski C, Manjin N, Rothbaum BO (2017). Virtual reality-enhanced extinction of phobias and post-traumatic stress. Neurotherapeutics.

[20] Sulway Shaleen, Whitney Susan L (2019). Advances in vestibular rehabilitation. Adv Otorhinolaryngol.

[21] Bergeron M, Lortie CL, Guitton MJ (2015). Use of virtual reality tools for vestibular disorders rehabilitation: a comprehensive analysis. Adv Med.

[22] Micarelli A, Viziano A, Micarelli B, Augimeri I, Alessandrini M (2019). Vestibular rehabilitation in older adults with and without mild cognitive impairment: effects of virtual reality using a head-mounted display. Arch Gerontol Geriatr.

[23] Micarelli A, Viziano A, Augimeri I, Micarelli D, Alessandrini M (2017). Three-dimensional head-mounted gaming task procedure maximizes effects of vestibular rehabilitation in unilateral vestibular hypofunction: a randomized controlled pilot trial. Int J Rehabil Res.

[24] Kiryu T, So RHY (2007). Sensation of presence and cybersickness in applications of virtual reality for advanced rehabilitation. J Neuroeng Rehabil.

[25] Öztürk Şeyma Tuğba, Şerbetçioğlu Mustafa Bülent, Ersin K, Yılmaz O (2021). The impact of optical illusions on the vestibular system. J Audiol Otol.

[26] Smyth N, Flynn M, Rajcani J, F Hucklebridge M, Thorn L, Wood C, Golding J, Evans P, Clow A (2019). Attenuated cortisol reactivity to psychosocial stress is associated with greater visual dependency in postural control. Psychoneuroendocrinology.

[27] Honaker JA, Kretschmer LW (2014). Impact of fear of falling for patients and caregivers: perceptions before and after participation in vestibular and balance rehabilitation therapy. Am J Audiol.

[28] Eagger S, Luxon LM, Davies RA, Coelho A, Ron MA (1992). Psychiatric morbidity in patients with peripheral vestibular disorder: a clinical and neuro-otological study. J Neurol Neurosurg Psychiatry.

[29] Balaban Carey D (2002). Neural substrates linking balance control and anxiety. Physiol Behav.

