# Supplementary material for: Appropriate Image Selection With Virtual Reality in Vestibular Rehabilitation: Cross-sectional Study
Source: JMIR Serious Games. 2023 Apr 13;11:e40806. doi: 10.2196/40806 (PMC10162482; doi:10.2196/40806)
Supplement: Multimedia Appendix 1 [file games_v11i1e40806_app1.pdf]

State-Trait Anxiety Inventory (STAI)

State - Trait Anxiety Inventory (STAI)

Name: **Age:** **Gender:** **Date:**

A number of statements which people have used to desribe themselves are given below. Read each statement and then circle the appropriate number to the right of the statement to indicate how you feel right now, that is, at this moment. There are no right or wrong answers. Do not spend too much time on any one statement but give the answer which seems to describe your present feelings best.

		NOT AT ALL	SOMEWHAT	MODERATELY SO	VERY MUCH SO
**1**	**I feel calm**	1 2 3 4	1 2 3 4	1 2 3 4	1 2 3 4
**2**	**I feel secure**	1 2 3 4	1 2 3 4	1 2 3 4	1 2 3 4
**3**	**I am tense**	1 2 3 4	1 2 3 4	1 2 3 4	1 2 3 4
**4**	**I feel strained**	1 2 3 4	1 2 3 4	1 2 3 4	1 2 3 4
**5**	**I feel at ease**	1 2 3 4	1 2 3 4	1 2 3 4	1 2 3 4
**6**	**I eel upset**	1 2 3 4	1 2 3 4	1 2 3 4	1 2 3 4
**7**	**I am presently worrying over possible misfortunes**	1 2 3 4	1 2 3 4	1 2 3 4	1 2 3 4
**8**	**I feel satisfied**	1 2 3 4	1 2 3 4	1 2 3 4	1 2 3 4
**9**	**I feel frightened**	1 2 3 4	1 2 3 4	1 2 3 4	1 2 3 4
**10**	**I feel comfortable**	1 2 3 4	1 2 3 4	1 2 3 4	1 2 3 4
**11**	**I feel self-confident**	1 2 3 4	1 2 3 4	1 2 3 4	1 2 3 4
**12**	**I feel nervous**	1 2 3 4	1 2 3 4	1 2 3 4	1 2 3 4
**13**	**I am jittery**	1 2 3 4	1 2 3 4	1 2 3 4	1 2 3 4
**14**	**I feel indecisive**	1 2 3 4	1 2 3 4	1 2 3 4	1 2 3 4
**15**	**I am relaxed**	1 2 3 4	1 2 3 4	1 2 3 4	1 2 3 4
**16**	**I feel content**	1 2 3 4	1 2 3 4	1 2 3 4	1 2 3 4
**17**	**I am worried**	1 2 3 4	1 2 3 4	1 2 3 4	1 2 3 4
**18**	**I feel confused**	1 2 3 4	1 2 3 4	1 2 3 4	1 2 3 4
**19**	**I feel steady**	1 2 3 4	1 2 3 4	1 2 3 4	1 2 3 4
**20**	**I Feel pleasant**	1 2 3 4	1 2 3 4	1 2 3 4	1 2 3 4
