# Supplementary material for: Appropriate Image Selection With Virtual Reality in Vestibular Rehabilitation: Cross-sectional Study
Source: JMIR Serious Games. 2023 Apr 13;11:e40806. doi: 10.2196/40806 (PMC10162482; doi:10.2196/40806)
Supplement: Multimedia Appendix 2 [file games_v11i1e40806_app2.pdf]

STAI FORM TX – I

İsim:.. Cinsiyet:.................

Yaş:.............. Meslek:.. Tarih:....../......./..........

YÖNERGE: Aşağıda kişilerin kendilerine ait duygularını anlatmada kullandıkları bir takım ifadeler verilmiştir. Her ifadeyi okuyun, sonra da o anda nasıl hissettiğinizi ifadelerin sağ tarafındaki parantezlerden uygun olanını işaretlemek suretiyle belirtin. Doğru ya da yanlış cevap yoktur. Herhangi bir ifadenin üzerinde fazla zaman sarf etmeksizin **anında** nasıl hissettiğinizi gösteren cevabı işaretleyin.

		HİÇ	BİRAZ	ÇOK	TAMAMİYLE
1.	Şu anda sakinim	(1)	(2)	(3)	(4)
2.	Kendimi emniyette hissediyorum	(1)	(2)	(3)	(4)
3	Su anda sinirlerim gergin	(1)	(2)	(3)	(4)
4	Pişmanlık duygusu içindeyim	(1)	(2)	(3)	(4)
5.	Şu anda huzur içindeyim	(1)	(2)	(3)	(4)
6	Şu anda hiç keyfim yok	(1)	(2)	(3)	(4)
7	Başıma geleceklerden endişe ediyorum	(1)	(2)	(3)	(4)
8.	Kendimi dinlenmiş hissediyorum	(1)	(2)	(3)	(4)
9	Şu anda kaygılıyım	(1)	(2)	(3)	(4)
10.	Kendimi rahat hissediyorum	(1)	(2)	(3)	(4)
11.	Kendime güvenim var	(1)	(2)	(3)	(4)
12	Şu anda asabım bozuk	(1)	(2)	(3)	(4)
13	Çok sinirliyim	(1)	(2)	(3)	(4)
14	Sinirlerimin çok gergin olduğunu hissediyorum	(1)	(2)	(3)	(4)
15.	Kendimi rahatlamış hissediyorum	(1)	(2)	(3)	(4)
16.	Şu anda halimden memnunum	(1)	(2)	(3)	(4)
17	Şu anda endişeliyim	(1)	(2)	(3)	(4)
18	Heyecandan kendimi şaşkına dönmüş hissediyorum	(1)	(2)	(3)	(4)
19.	Şu anda sevinçliyim	(1)	(2)	(3)	(4)
20.	Şu anda keyfim yerinde.	(1)	(2)	(3)	(4)
